# Lung Inflammation in alpha-1-antitrypsin deficient individuals with normal lung function

**DOI:** 10.1186/s12931-023-02343-3

**Published:** 2023-02-02

**Authors:** Nurdan Kokturk, Nazli Khodayari, Jorge Lascano, E. Leonard Riley, Mark L. Brantly

**Affiliations:** 1 Division of Pulmonary, Critical Care and Sleep Medicine, J. Hillis Miller Health Science Center, University of Florida College of Medicine, P.O. Box 100225, Gainesville, FL 32610-0225 USA; 2 Department of Pulmonary and Critical Care, Gazi University School of Medicine, Ankara, Turkey; 3 Kansas City Veterans Affair Medical Center, Kansas, TX USA

**Keywords:** Epithelial lining fluid, Neutrophil elastase, Protease inhibitor, Pulmonary function

## Abstract

**Background:**

Alpha-1-antitrypsin deficient (AATD) individuals are prone to develop early age of onset chronic obstructive pulmonary disease (COPD) more severe than non-genetic COPD. Here, we investigated the characteristics of lower respiratory tract of AATD individuals prior to the onset of clinically significant COPD.

**Methods:**

Bronchoalveolar lavage was performed on 22 AATD with normal lung function and 14 healthy individuals. Cell counts and concentrations of proteases, alpha-1-antitrypsin and proinflammatory mediators were determined in the bronchoalveolar lavage fluid from study subjects. In order to determine the airway inflammation, we also analyzed immune cell components of the large airways from bronchial biopsies using immunohistochemistry in both study subjects. Finally, we made comparisons between airway inflammation and lung function rate of decline using four repeated lung function tests over one year in AATD individuals.

**Results:**

AATD individuals with normal lung function had 3 folds higher neutrophil counts, 2 folds increase in the proteases levels, and 2–4 folds higher levels of IL-8, IL-6, IL-1β, and leukotriene B4 in their epithelial lining fluid compared to controls. Neutrophil elastase levels showed a positive correlation with the levels of IL-8 and neutrophils in AATD epithelial lining fluid. AATD individuals also showed a negative correlation of baseline FEV_1_ with neutrophil count, neutrophil elastase, and cytokine levels in epithelial lining fluid (p < 0.05). In addition, we observed twofold increase in the number of lymphocytes, macrophages, neutrophils, and mast cells of AATD epithelial lining fluid as compared to controls.

**Conclusion:**

Mild inflammation is present in the lower respiratory tract and airways of AATD individuals despite having normal lung function. A declining trend was also noticed in the lung function of AATD individuals which was correlated with pro-inflammatory phenotype of their lower respiratory tract. This results suggest the presence of proinflammatory phenotype in AATD lungs. Therefore, early anti-inflammatory therapies may be a potential strategy to prevent progression of lung disease in AATD individuals.

**Supplementary Information:**

The online version contains supplementary material available at 10.1186/s12931-023-02343-3.

## Background

Alpha1-antitrypsin (AAT) is the major plasma serine protease inhibitor, playing an important role in limiting tissue injury mediated by proteases during inflammation. Alpha1-antitrypsin deficiency (AATD) is an inherited monogenic disorder associated with reduced circulating levels of AAT, linked to increased susceptibility to chronic obstructive lung disease (COPD) [1, 2]. COPD, one of the top five leading causes of death in the world, is a destructive lung condition with a slow progression [3–6]. AATD Individuals with COPD have a Two-to-eightfold increased rate of decline in FEV_1_ (∆FEV_1_) as compared to non-genetic COPD individuals [7–11].

AAT is an acute phase protein mainly produced by the liver, and circulating concentrations of AAT increase two to four fold during inflammation. The function of AAT is described as neutralization of proteases such as neutrophil elastase (NE) in the lung’s lower respiratory tract of the lung [12]. NE is the major serine protease released by activated neutrophils and the main substrate for AAT inhibitory function. Increased numbers of neutrophils, have been reported in the lower respiratory tract of AATD individuals with COPD, contributing to lung destruction [12, 13]. Lung low levels of AAT therefore contribute to increased activity of proteases and are thought to be a major cause for the development of AATD lung disease [14].

Chronic inflammation of the lower respiratory tract due to inhalation of cigarette smoke (CS) is the major cause of COPD [15, 16]. Exposure to dust [17–19], and infections [20] are also associated with an increased rate of lung function decline in COPD patients. CS stimulates lung resident immune cells to release pro-inflammatory mediators that enhance neutrophil’s recruitment [21, 22]. During inflammation, activated neutrophils arrive in the lung via a cytokine gradient to provide host defense releasing proteases such as NE, proteinase 3 (PR3) [23, 24]. An increase in airway neutrophil recruitment in COPD correlates with the lung function decline [25]. NE induces the production of pro-inflammatory cytokines and inactivates extracellular immune mediators such as complement components in favor of inflammation [26]. NE also increases vascular permeability that amplify lung inflammation and tissue damage during inflammation [27]. As an anti-inflammatory molecule, AAT provides protection against NE cytotoxicity and therefore insufficient AAT, in combination with increased lung recruitment of neutrophils, resulting in the development of AATD lung injury [28].

Increased pro-inflammatory mediators have been showed in the sputum and bronchoalveolar lavage fluid (BALF) from AATD individuals with COPD, indicating presence of airway inflammation [29–31]. Therefore, it is reasonable to believe that the intensity of inflammation correlates with the proteolytic environment and inflammatory mediators’ burden in AATD lungs. However, little is known about the temporal appearance of inflammation and neutrophil’s recruitment, in the lungs of AATD individuals. Here, we focused on immune cells and pro-inflammatory mediators in the lower respiratory tract of AATD individuals with normal lung function to explore the temporal inflammatory changes in AATD lungs. The cellular profile of BALF and immunohistochemistry analysis of bronchial biopsy samples showed the pre-inflammatory phenotype of AATD lungs as compared to control subjects. Furthermore, investigation of the levels of inflammatory mediators in the BALF and their association with lung function rate of decline suggested a declining trend in the lung function of AATD individuals which was correlated with pro-inflammatory phenotype of lower respiratory tract. Given these findings, we speculate that early anti-inflammatory therapies may prevent further progression of lung disease in individuals with AATD.

## Methods

### Study population

Normal and AATD individuals were recruited at the NIH Clinical Center after providing consent for the NIH IRB protocol # 95-H-0016. All individuals were free of any signs of respiratory tract infection at the time of study. All BALF samples were screened using clinical laboratory methods for common viral and bacterial pathogens. AAT PI-typing was determined by isoelectric focusing of the serum proteins and AAT serum levels were determined by nephelometry (Behring Diagnostics, Marburg, Germany) using in-house standards and controls [32].

### Lung function tests

AATD individuals were evaluated every three months for one year, and pulmonary function tests were performed according to American Thoracic Society (ATS) standards [33]. The post-bronchodilator FEV_1_ for each individual was used to calculate the rate of decline for FEV_1_ expressed in ml per year ($\Delta$FEV_1_).

### Bronchoalveolar lavage fluid

All bronchoscopies were performed by at the NIH Clinical Center, Bethesda MD, USA as previously described [34, 35]. A flexible video bronchoscope was inserted through the mouth via a mouthpiece with the subject. Instillation of 100 mL of saline was performed in 5 sequential 20 mL aliquots for each of the three lobes (typically right middle medial, right upper anterior and lingula superior or inferior, 300 mL in total). The aliquots from each lobe were collected separately in 50 mL conical tubes and stored on ice until processed.

### Determination of antigenic AAT level in ELF (Epithelial Lining Fluid)

AAT concentration in BALF was determined by indirect sandwich ELISA. Briefly, Immulon-2 plates (Dynatech, Chantilly, VA Cat # 112079, 81079) were coated overnight with goat anti-human AAT antibody (ICN, Costa Mesa, CA. Cat # 855111). Plates were washed, and samples and standards were added. Purified AAT from normal plasma was used to generate a standard curve [32]. The plates were washed and incubated at with rabbit anti-human AAT antibody (Dako, Santa Clara, CA. Cat # A0012) followed by incubation with horseradish peroxidase conjugated goat anti-rabbit IgG (BioRad. Hercules, CA. Cat # 1706515). Finally, o-phenylendiamine-dihydrochoride (Fluka Biochemika, Milwaukee, WI. Cat # R1413) substrate was added to develop the ELISA. For each subject, an ELF dilution factor was calculated by taking the ratio of their plasma urea concentration to BALF urea concentration. BALF measurements are multiplied by this factor to standardize ELF measurements.

### Determination of functional AAT levels in BALF (anti-NE capacity assay)

All BALF samples were treated with 4 M methylamine (Sigma, St.Louis, MO. Cat # 534102) at room temperature for 1 h. Standards and samples were added to the plates and were incubated at 37 °C for 5 min. Human NE was added and incubated for 5 min (Athens Research and Technology Inc., Athens, GA. Cat # 16–14-051200). Methoxysuccinyl-ala-ala-val-pro-p-nitroanilide (Sigma, St. Louis, MO. Cat # M4765) was added and kinetic optical density at 405–490 nm was determined.

### Determination of AAT:NE complex concentrations in BALF

Plates were coated with sheep anti-human NE (ICN, Costa Mesa, CA. Cat # LS-C23012-200) and incubated at 4 °C overnight. Plates were washed and samples and standards were loaded for incubation at 37 °C for 1.5 h. Rabbit anti-AAT antibody was added and incubated at 37 °C for 1.5 h. The plates were then washed and horseradish peroxidase conjugated goat anti-rabbit IgG was added. Reactions were terminated by H_2_SO_4_, and the optical density at 490 nm was determined. Values were corrected for BALF dilution to give ELF concentrations of AAT:NE complexes.

### Determination of antigenic NE concentrations

Samples and standards pre-treated with 1 mM PMSF (Sigma, St. Louis, MO. Cat # P7626) were loaded to the sheep anti-human NE coated plates and were incubated at 37 °C for 1 h. Rabbit anti-NE (Athens Research and Technology, Athens, GA. Cat # 01-14-051200) was added and incubated for 1 h 37 °C. The plates were washed and horseradish peroxidase conjugated goat anti-rabbit IgG was added. Reactions were terminated by adding H_2_SO_4_, and optical density at 490 nm was determined using a SPECTRAmax (Molecular Devices, Sunnyvale, CA). Values were corrected for BALF dilution to give ELF concentrations of NE.

### Determination of inflammatory mediators in BALF

Cytokine levels were measured using R&D Systems ELISA kits (Minneapolis, MN) according to the manufacturer’s instructions. Briefly, samples were diluted in assay buffer before being applied to the assay plates for incubation. The plates were then washed and conjugated antibodies applied for the second incubation. Plates were washed and substrate was added. The optical density was measured and sample concentrations were calculated based on the standard curve.

### Bronchial biopsies

Bronchial biopsies were taken from the carinas of the right lower lobe (RLL) and right middle lobe (RML) using a small cut biopsy forceps as previously described [36]. A maximum of 5 biopsies per individual were taken. Biopsies were stored in 10% formalin for further evaluation. Immunohistochemical analysis was performed on a subset of the 11 AATD individuals (6 males and 5 females) and 6 control volunteers (4 males and 2 females).

### Tissue processing and immunohistochemistry (IHC)

Tissues were fixed in formalin and following standard paraffin embedding procedures, 4 µm sections were collected onto clean glass slides. The tissue sections were deparaffined with xylene and rehydrated. To study the airways histological features, sections were stained with hematoxylin and eosin. IHC was used to determine presence of inflammatory cells. Primary antibodies against leukocyte markers, including anti-human NE (ICN, Costa Mesa, CA. Cat # LS-C23012-200) (neutrophils), anti-human CD68 (ThermoFischer Scientifics, Waltham, MA. Cat # MA5-12407) (macrophages), anti-human CD45R0 (ThermoFischer Scientifics, Waltham, MA. Cat # MA5-11532) (T lymphocytes), anti-human CD3 (ThermoFischer Scientifics, Waltham, MA. Cat # MA5-12577) (T lymphocytes), anti-human CD4 (ThermoFischer Scientifics, Waltham, MA. Cat # MA5-32166) (T-helper cells), anti-human CD8 (ThermoFischer Scientifics, Waltham, MA. Cat # MA5-14584) (cytotoxic T cells), anti-human CD20 (Abcam, Waltham, MA. Cat # ab78237) (B cells), anti-human EG2 (Abcam, Waltham, MA. Cat # EPR20357) (eosinophils), and anti-human AA1 (Abcam, Waltham, MA. Cat # ab2378) were used. The dilution factor for the antibodies were according to the manufacturer’s instructions. The sections were incubated with 3% H_2_O_2_ in 95% alcohol for 10 min to block endogenous peroxidase activity and primary antibody solutions were applied for antigen detection. Positive (tonsil and lymph node sections) and negative (no primary antibody) controls were run in each experiment.

### Airway biopsy image analysis

Cell counts were carried out by using a light microscope at a magnification of 40× using the Image-ProPlus Imaging software (Media Cybernetics, Inc. Rockville, MD). Positive cells were counted in representative areas that were subtended by 100 µm of intact basement membrane and that extended 100 µm into the submucosa. Counting was performed at locations where the thickness of submucosa was more then 100 µm and smooth muscle and mucosal glands were absent. A grid system was set up to select areas correctly. Each site in each square in the grid was 100 µm. 10 of those squares, which met the above criteria, were counted. The cumulative count was expressed as the number of the positive cells per mm of BM (cells/mm^2^).

### Other measurements

The LTB4 measurements and determination of ELF volume were performed as described previously [24]. Samples were treated with ethanol, centrifuged at 375 ×*g* for 10 min, acidified, and applied to C18 cartridges at a rate of 0.5 ml/minute. The C18 columns were sequentially washed using distilled water, 10% ethanol, and petroleum ether before elution of the sample using methyl-formate (Sigma, St.Louis, MO. Cat # 291056). The methyl-formate was dried under nitrogen gas and the sample was suspended in assay buffer. LTB4 was measured using an ELISA kit (Cayman Chemical. Ann Arbor, MI. Cat # 5220111).

### Statistical analysis

We extracted data on sample size, mean cytokine concentration, standard deviation (SD), and p-value to calculate the effective size. Statistical analysis was performed using GraphPad Prism 9 software (San Diego, CA). The results are shown as mean ± SEM unless otherwise stated. p-value of < 0.05 was considered statically significant. Data were tested for normal distribution using the Kolmogorov–Smirnov test. For the comparison of cell counts, proteases, and inflammatory mediators between the disease and control groups, student *t* test and Mann–Whitney U test were performed. The Spearman’s correlation coefficient was calculated to analyze the correlation between inflammatory mediators, proteases and the lung function test results. The "r value" is being used to indicate the correlation value referring to the Pearson correlation.

## Results

### Profile of study subjects

The study population consisted of 22 AATD individuals (9 male, 13 female) with a mean serum AAT level of 5.2 $\pm$ 0.2 μM. Of the AATD group, 8 were ex-smokers, with average history less than a 5 pack/year and quited smoking at least one year before the study. The remaining 14 were never smokers. No AATD patients were on AAT replacement therapy. We also recruited 14 healthy non-smoking homozygous PiMM controls (10 male, 4 female), with mean serum AAT levels of 27.7 $\pm$ 1.0 μM. Except for the serum AAT level, the demographic and clinical characteristics were similar between the two groups (Table 1).

**Table 1:** Study Population Characteristics

Study	Deficient	Normal	p value
Age (years)	42 + 2	40 + 4	0.17
FEV_1_ (% predicted)	101 + 3	107 + 4	0.058
FVC (% predicted)	103 + 6	104 + 4	0.43
TLC (% predicted)	105 + 4	104 + 4	0.82
DLco^a^ (% predicted)	112 + 4	103 + 5	0.22
$\Delta$FEV_1_ (ml/year)	− 174 + 59	NA	NA
$\alpha$_1_-AT Level ($\upmu$M)	5.2 + 0.2	27.7 + 1.0	< 0.0001^b^

Values are expressed as mean and standard error of the mean (SE)

FEV_1_, forced expiratory volume in one second; FVC, forced vital capacity; TLC,  total lung capacity; DL_CO_, diffusing capacity; $\Delta$ FEV_1_, rate of decline FEV_1_/year

^a^Single breath (uncorrected)

^b^Significant correlation

### BALF cellular features

We examined BALF cellular features every three months, over a one-year period. We found no significant differences in the cellular and biochemical features of four sequential four BALF samples from AATD subjects, therefore only the first BALF results are presented. We also observed no significant differences in the features of the three lobes and the data are reported from a single lobe, typically the right middle lobe, for simplicity. There were no differences in the ELF volume, total cells of the recovered ELF fluid, as well as percent of macrophages or lymphocytes in BALF between AATD and normal individuals (Table 2). By contrast we observed a significant increase in the ELF number of neutrophils (Additional file 1: Fig. S1A) in AATD individuals compared to healthy controls. The ELF percentage of neutrophils of AATD individuals was persistently increased and did not significantly vary over the one year of the experiment duration (Additional file 1: Fig. S1B).

**Table 2:** Bronchoalveolar lavage fluid features in alpha1-antitrypsin deficient individuals with normal pulmonary function and normal individuals

	Deficient	Normal	p-value
Return^a^	50.1 + 3.9	56.8 + 4.2	0.88
Total cells (ml^−1^ ELF)	1.4 × 10^7^ + 0.25 × 10^7^	1.2 × 10^7^ + 0.19 × 10^7^	0.25
Macrophages (%)	89.6 + 0.9	91.1 + 1.1	0.59
Neutrophils (%)	3.3 + 0.6	1.0 + 0.1	0.006^b^
Lymphocytes (%)	6.6 + 1.0	7.8 + 1.1	0.20
ELF volume (mL)	0.89 + 0.13	0.93 + 0.11	0.74

Values are expressed as mean and standard error of the mean (SE)

*ELF* epithelial lining fluid

^a^Percentage of BALF recovered

^b^Significant difference

### ELF inflammatory mediators

**Fig. 1:**
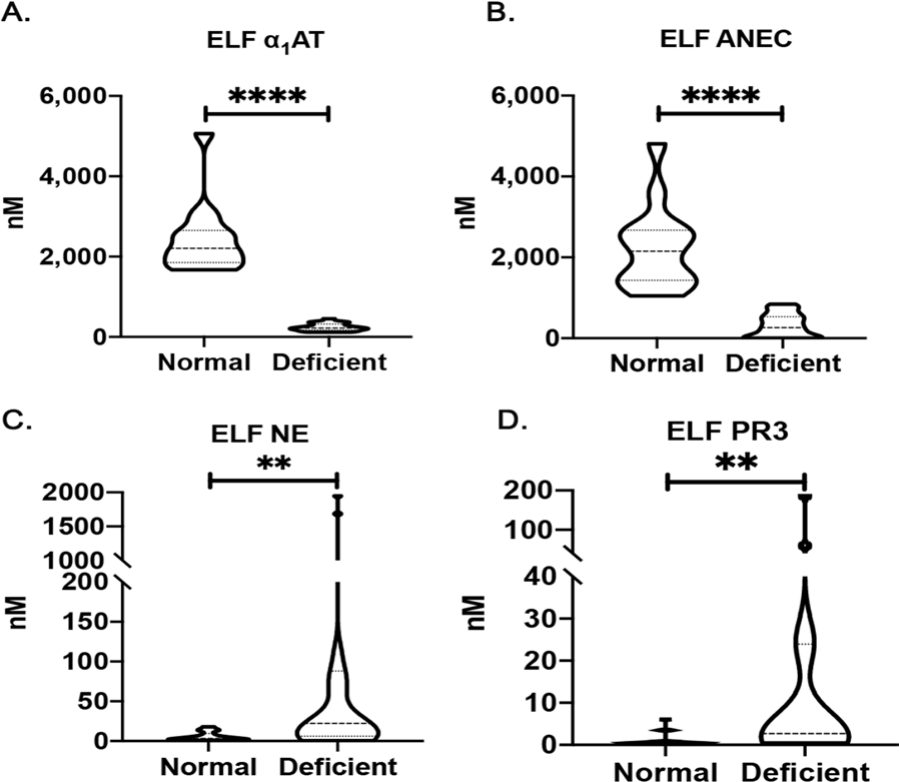
Protease-antiprotease imbalance in the epithelial lining fluid (ELF) of alpha1- antitrypsin deficient individuals. Bronchoalveolar lavage was performed in control subjects (N = 14), and alpha1- antitrypsin deficient (N = 22) individuals as described in the methods. **A** The concentrations of alpha1- antitrypsin, **B** Alpha1- antitrypsin:anti-neutrophil elastase capacity (ANEC), **C** neutrophil elastase (NE), and **D** protease 3 (PR3) were measured by ELISA and expressed as levels of analyte in the ELF. **P < 0.005, ****P < 0.00005

ELISA showed tenfold higher AAT concentrations in the ELF of normal individuals compared with AATD, as expected (Fig. 1A). There were higher levels of functionally active AAT within the ELF of normal subjects compared to AATD individuals (anti-neutrophil elastase capacity, ANEC) (Fig. 1B). Median NE concentration and PR3 levels within the ELF of AATD individuals were also higher than controls (Fig. 1C and D). There were no differences in the concentration of NE-AAT complexes in the ELF of AATD and normal volunteers due to low levels of AAT in AATD group and low levels of NE in the normal controls (Additional file 1: Fig. S1C).

**Fig. 2:**
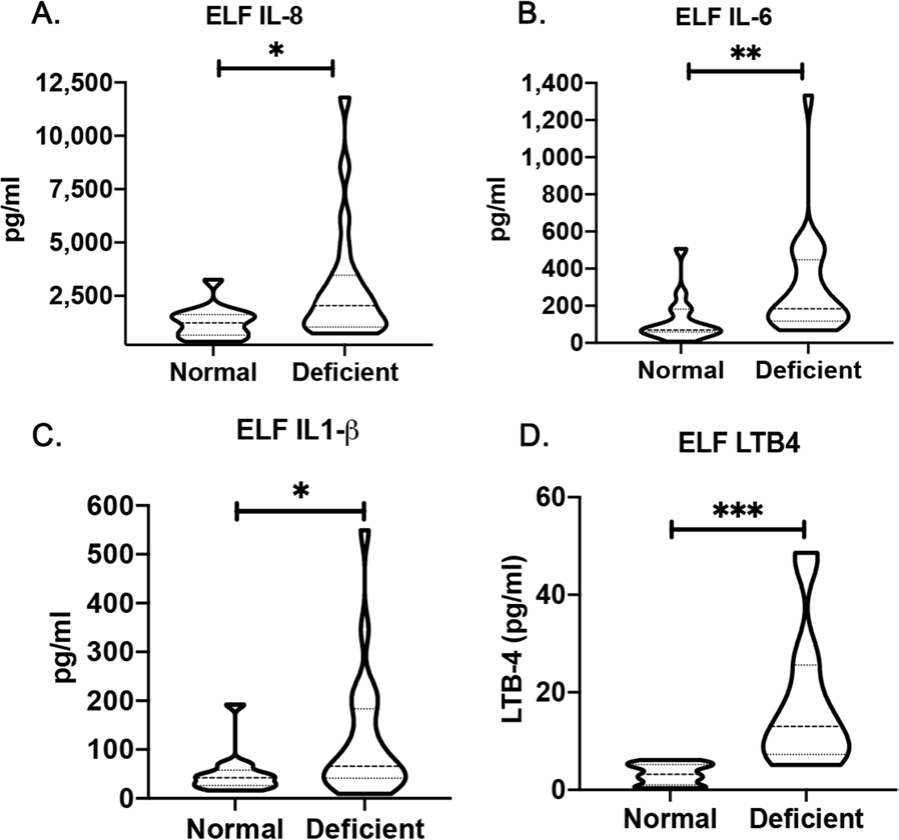
Increased pro-inflammatory mediators in the epithelial lining fluid (ELF) of alpha1- antitrypsin individuals. The concentration of pro-inflammatory cytokines was measured by ELISA and expressed as pg/ml levels of analyte in the ELF. **A** IL-8; **B** IL-6; **C** IL-1β; and **D** LTB4. *P < 0.05, **P < 0.005, and ^***^P < 0.0005

Next, we measured the concentrations of IL-8, IL-6, IL-1 beta and LTB4 in the BALF samples and found that AATD individuals had significantly greater levels of the pro-inflammatory mediators than controls (Fig. 2A–D) including a sixfold greater ELF concentration of LTB4 in AATD individuals.

### Lower respiratory tract inflammation

**Fig. 3:**
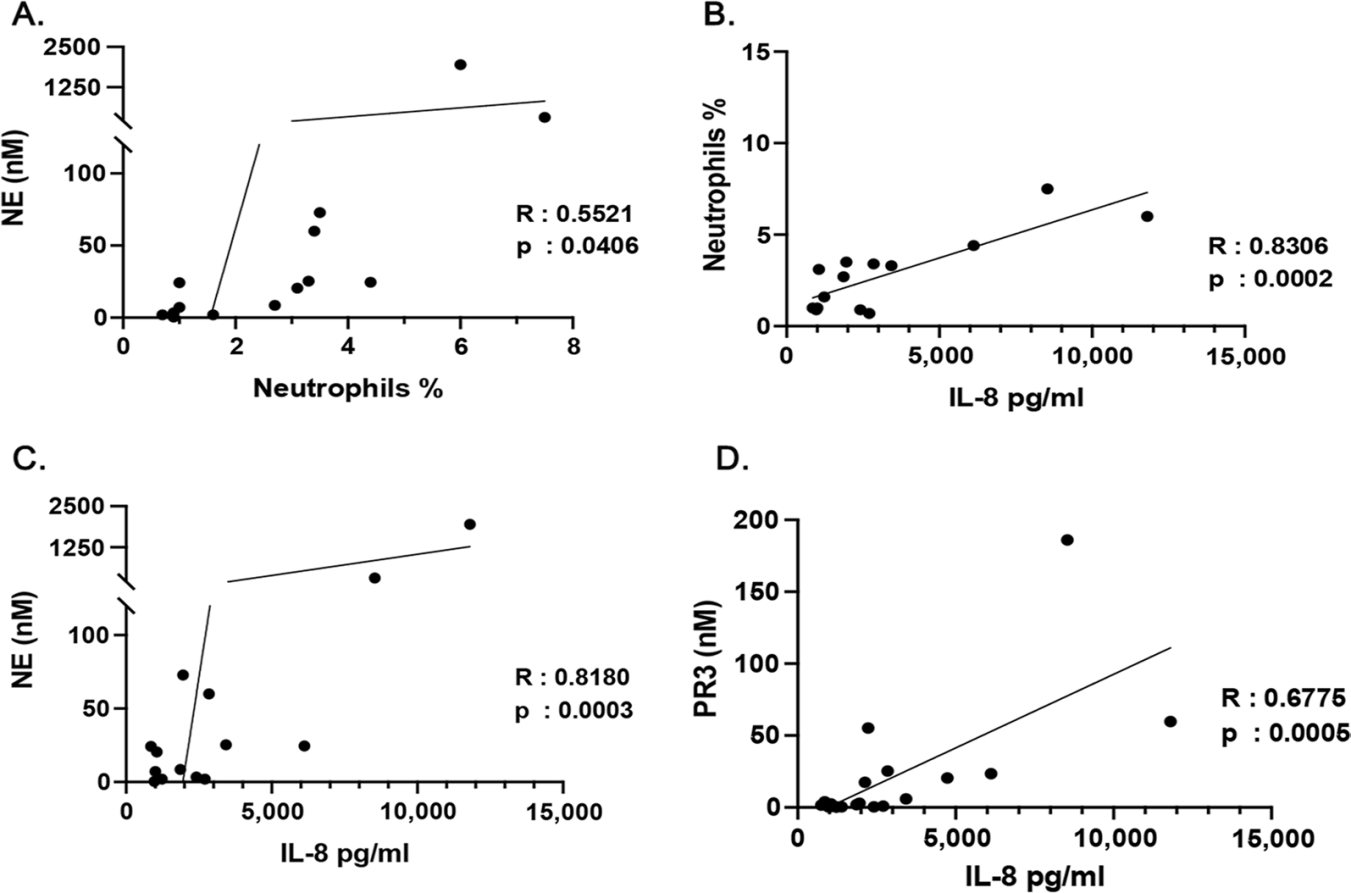
Correlation of pro-inflammatory mediators in the epithelial lining fluid (ELF) of alpha1- antitrypsin deficient individuals with neutrophils and neutrophil derived proteases. **A** The correlation between the percentage of neutrophils and neutrophil elastase concentrations (NE), and **B** the correlation between neutrophil percentage and the levels of IL-8 in the ELF of alpha1- antitrypsin subjects. **C** The correlation between the levels of neutrophil elastase and IL-8 in the ELF of alpha1- antitrypsin subjects. **D** The correlation between protease 3 (PR3) concentration and the levels of IL-8

Our correlation studies indicated a positive correlation between the percentage of neutrophils and NE concentrations in the ELF of AATD individuals (Fig. 3A). Importantly, the percentage of neutrophils and the levels of NE also showed a positive correlation with IL-8 levels in the lower respiratory tract (Fig. 3B and C), whereas neither IL-1beta nor IL-6 correlated with NE concentration. Similarly, the levels of IL-8 in the lower respiratory tract were also positively correlated with the levels of PR3 (Fig. 3D).

**Fig. 4:**
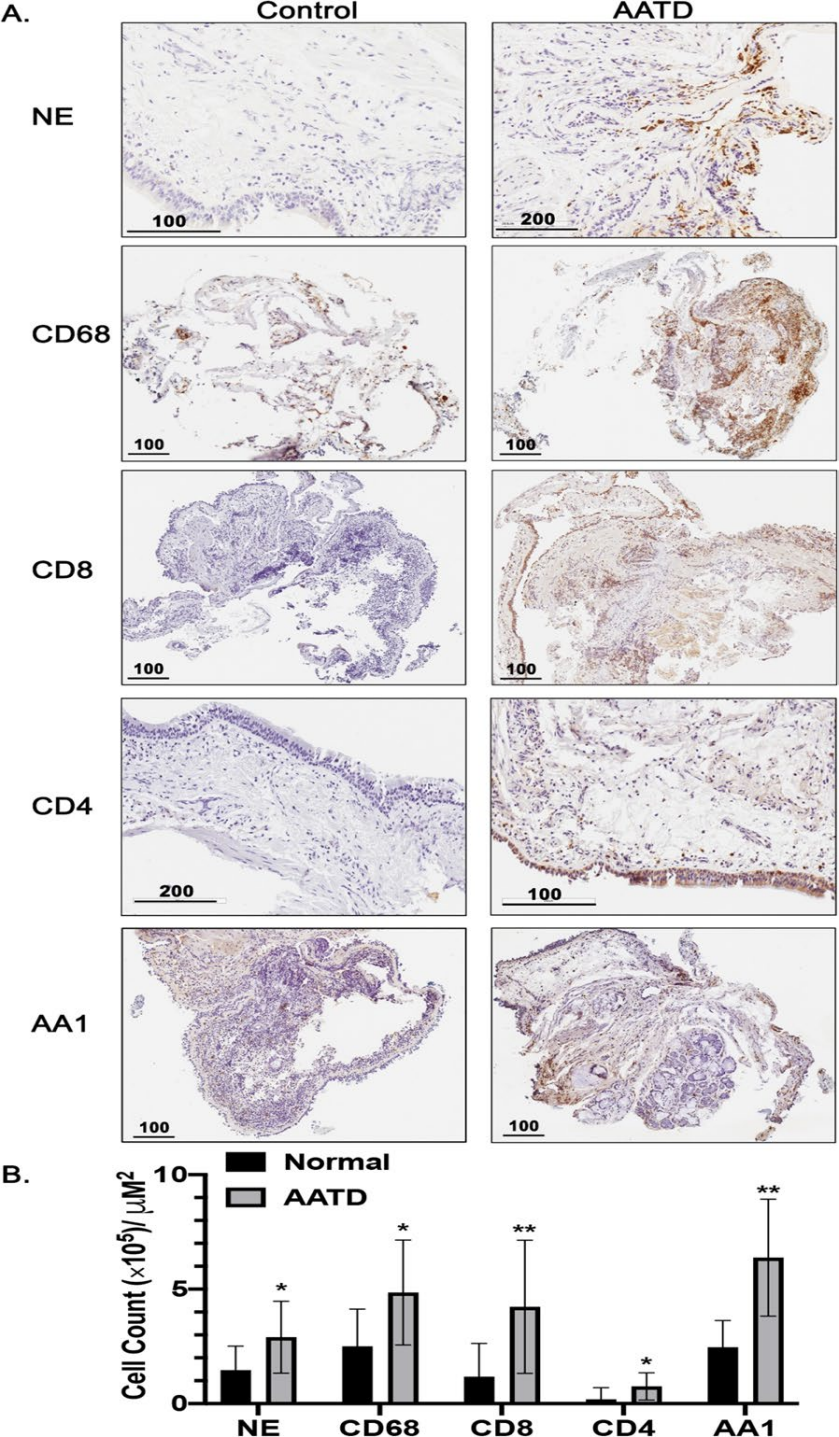
Large airway inflammatory cells in normal and alpha1- antitrypsin deficient Individuals. **A** Bronchial biopsies from normal and alpha1- antitrypsin deficient individuals were incubated with primary antibodies against CD68, CD45, CD8, neutrophil elastase (NE), and AA1; HRP-conjugated secondary antibody and HRP-DAB developer as described in the methods. **B** The cells were counted and expressed as the number of cells per mm2 of basement membrane in the section. * P < 0.05, ** P < 0.005

Furthermore, IHC analysis of the bronchial biopsies showed higher neutrophils and CD68^+^ macrophages in AATD airways compared to controls. Additionally, there were also an increased numbers of CD8^+^ and CD4^+^ T cells, and AA1^+^ mast cells in the bronchial epithelium of AATD individuals (Fig. 4A and B).

### Lung function

**Fig. 5:**
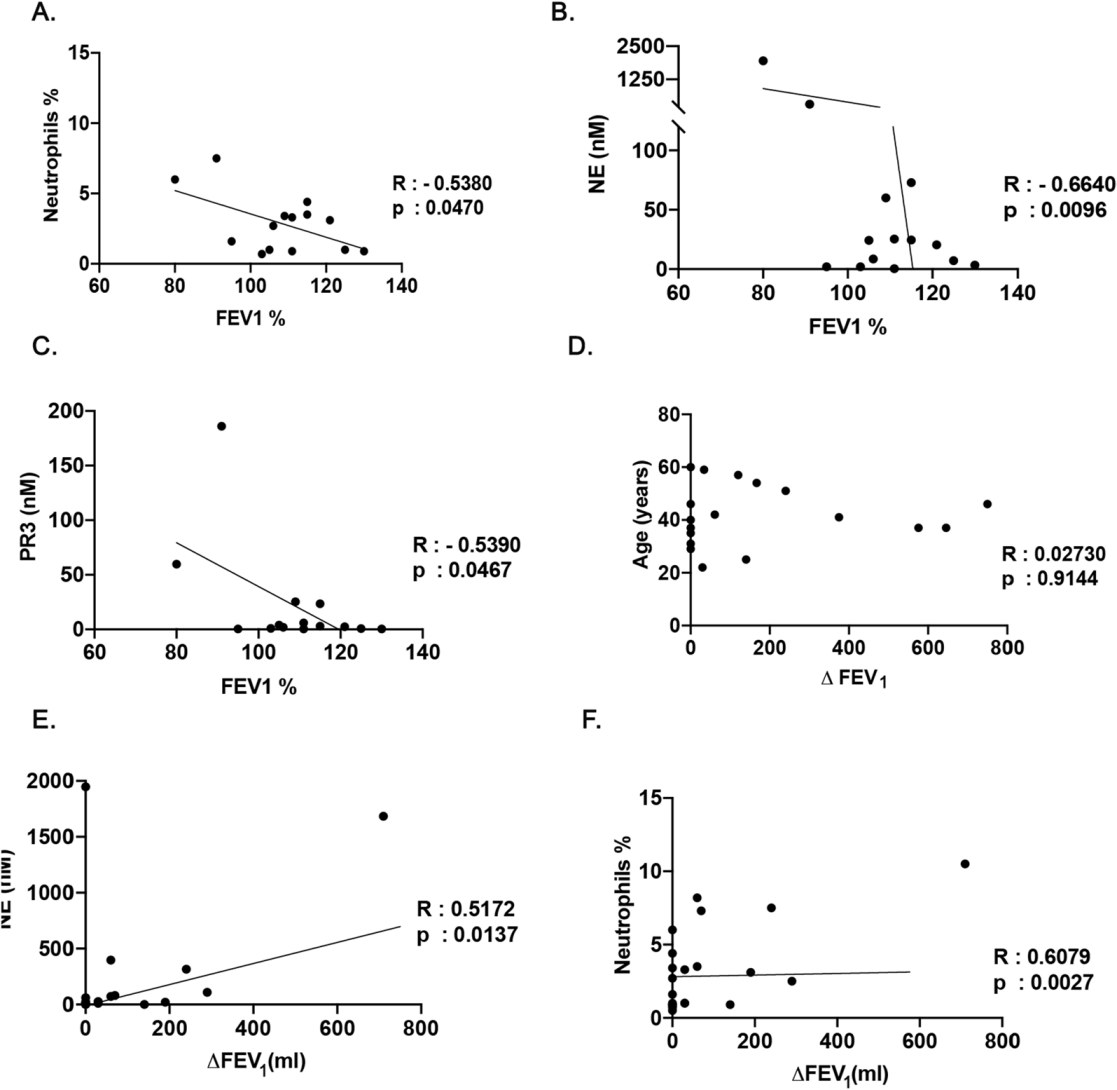
Correlation of lung function rate of decline and inflammatory state of the lower respiratory tract in alpha1- antitrypsin deficient individuals. **A** The correlation of initial FEV1 with the percentage of neutrophils, **B** the concentration of neutrophil elastase, and **C** the concentration of protease 3 (PR3) in the lower respiratory tract of alpha-1-antitrypsin deficient individuals. **D** The correlation of the rate of decline in lung function ($\Delta$ FEV1) with age of individuals α-1 antitrypsin deficiency, and **E** the concentration of neutrophil elastase, **F** as well as percentage of the neutrophils of the lower respiratory tract in the individuals with alpha1- antitrypsin deficiency

To determine the relationship between the inflammatory state of the AATD lower respiratory tract with lung function rate of decline, we examined FEV_1_ values over a one-year period. Initial FEV_1_ showed a negative correlation with the percentage of neutrophils (Fig. 5A), NE and PR3 levels in the lower respiratory tract of AATD individuals (Fig. 5B and C). Furthermore, our results also indicated that $\Delta$FEV_1_ does not correlate with age in AATD individuals (Fig. 5D). Importantly, $\Delta$ FEV_1_ was significantly correlated with NE levels (Fig. 5E), and the percentage of neutrophils (Fig. 5F) in the AATD lower respiratory tract.

## Discussion

Approximately 1:120 COPD patients, have the disease secondary to AATD [37]. While some studies indicate that AATD individuals may live a normal life span with little functional lung impairment [28], others may develop progressive disease, even in the absence of known risk factors [38]. However, it has been unclear when the AATD-mediated lung inflammatory phenotype starts to develop and how early it is clinically detectable. To answer this question, we evaluated an AATD cohort who presented normal lung function, using a protocol that included lung function tests, bronchoalveolar lavage fluid, bronchial biopsies, and 4 visits over a year. This protocol allowed us to explore relationships between pulmonary inflammation, immune cell infiltration and lung function in our study subjects. Here, we illustrate that AATD individuals have higher neutrophil counts and levels of NE, IL-8, IL-6, IL-1β, and leukotriene B4 in their epithelial lining fluid prior to the onset of symptomatic lung destruction. The levels of proteases shows a positive correlation with the levels of IL-8 and neutrophils in AATD epithelial lining fluid. We also observed that there is a negative correlation between the baseline FEV_1_ and neutrophil counts, neutrophil elastase, and cytokine levels in epithelial lining fluid of AATD individuals. Our results also show an increase in the number of lymphocytes, macrophages, neutrophils, and mast cells of AATD epithelial lining fluid as compared to control subjects.

Lungs have been shown to serve as a target organ for airborne pathogens and allergens that cause inflammation due to continuous exposure [39]. Previous studies have reported that air pollution, and inhaled particulates stimulate neutrophils infiltration within the airways [40] and increase AAT levels [41], resolving within days in normal individuals [42]. In contrast, and consistent with our results, there is an increased risk of lung damage associated with neutrophil infiltration in AATD individuals, due to low levels of AAT and unopposed activity of proteases regardless of CS exposure [43]. Furthermore, CS causes lung neutrophilic inflammation which is expected to be larger in AATD smokers than in smokers with normal AAT levels [44]. Neutrophilic inflammation in AATD airways could be exacerbated as a result of AAT polymers deposited in alveolar and bronchial epithelial cells, acting as potent chemoattractants for neutrophils [36]. Previous studies have shown that CS polymerizes AAT, exacerbating inflammation [45]. Therefore, our results may also support the notion that AATD lung resident cells including alveolar macrophages and epithelial cells play a role in early AATD lung inflammation due to toxic gain of function, analogous to hepatocytes. In this regard, we and others have shown that AATD macrophages with AAT accumulation have impaired efferocytosis, and activation of the unfolded protein response, and spontaneously produce pro-inflammatory cytokines [37–39]. Overall, these findings suggest that early lung inflammation due to a lack of AAT might be part of the mechanism for the progression of AATD lung disease.

NE has been shown to increase the production of LTB4 by macrophages which is a potent neutrophil chemoattractant. LTB4 prompts macrophages to secrete pro-inflammatory cytokines in an autocrine manner [46]. Our data reveal higher concentrations of LTB4, as well as IL-6, IL-8, and IL-1beta in the ELF of AATD individuals with normal lung function compared to healthy controls. A similar observation was also made in sputum from AATD individuals with severe lung function impairment by Woolhouse et al. [40]. In agreement with these findings, we observed that higher levels of IL-8 positively correlated with the number of neutrophils, and the levels of NE and PR3 in the ELF of AATD individuals with normal lung function. IL-8, an important chemoattractant [47], can additionally activate neutrophils to release proteases. Likewise, it has been shown that IL-8 causes physiological changes in neutrophils during inflammation [47]. Therefore, IL-8 derived neutrophilic inflammation is a fundamental mechanism for pathogenesis of many of the lung diseases [48]. Our observation confirms an increase in neutrophils and proteases that correlate with the levels of IL‐8 in AATD lungs, despite lack of clinical symptoms. This suggests a further contribution of early airway inflammation to the development of the lung deterioration observed in AATD individuals later in life.

AATD individuals with significant lung disease have excessive inflammatory cells in the lungs [13], However, it is unclear when this inflammatory process begins [20]. Here, we observed an increased number of inflammatory cells in the bronchi of AATD individuals with normal lung function compared to the controls. This increased number of cells including CD4^+^ and CD8^+^ lymphocytes, is consistent with the report from Baraldo, et al. indicating increased lymphocytes in the lungs of AATD individuals with severe emphysema [49]. Our results also indicated increased number of neutrophils, macrophages, and mast cells in the bronchi of AATD individuals with normal lung function. Therefore, we believe infiltration of inflammatory cells to the airway epithelium happens secondary to the inflammatory environment of the AATD airways, and prior to the onset of clinically significant lung function impairment. This early abnormal presence of inflammatory cells perpetuate the inflammatory niche, and may explain the hyper-responsiveness and progressive lung damage in AATD individuals.

Chronic airway inflammation plays a central role in the pathophysiology of COPD and is associated with an accelerated decline in FEV_1_ [50]. In AATD individuals with COPD, a negative correlation between initial FEV_1_ and ELF neutrophil burden has been reported [51]. However, it is unclear whether increased neutrophils have a causal relationship with AATD lung function decline. The contribution of proteases to the etiology of airway obstruction prior to the development of severe AATD lung destruction is even less well established. Here, we show an unexpected negative correlation between initial FEV_1_ and neutrophil burden in the ELF of AATD individuals. According to previous reports, ∆ FEV_1_ levels accelerate as age increases [52]. In agreement with previous reports [53] measuring the FEV_1_ during 1 year of follow-up, we observed that in AATD individuals, ∆ FEV_1_ was independent of age and negatively correlated with the ELF levels of NE and neutrophil burden. The correlation between increased neutrophils and immunomodulators of the lower respiratory tract with ∆FEV_1_ suggests a role for early persistent inflammation in the development of lung injury prior to AATD lung function impairment. This observation indicates of a baseline ongoing airway inflammation in AATD individuals, without symptoms or clinical evidence of lung disease.

Our study has several limitations, the sample size is small and is only limited to subjects with normal lung function. It will be important to confirm the relationship between inflammation and lung function in larger cohorts of AATD subjects at different stages of disease and lung function. We also included ex-smokers in our AATD cohort. Even though it was minimal and remote smoking history it could affect our results. Finally we did not have chest imagen to evaluate potential emphysematous changes despite normal lung function. Chest CT densitometry might also be helpful for the early detection of AATD lung destruction [54].

## Conclusions

In conclusion, this study supports a multitude of AAT biological functions including modulation of several pathogenic processes underlying lung destruction. We demonstrate that normal environmental exposures might not only lead to a consistent airway inflammation, but also lead to continued infiltration of inflammatory cells within the AATD airways prior to the clinical evidence of lung disease. Our results suggest that AATD lung inflammation prior to significant lung damage might be modulated by early treatment with AAT replacement therapy and other anti-inflammatory therapies. Furthermore, our data also provide further rationale for the use of NE inhibitors in AATD as well as other lung diseases associated with NE-induced inflammation.

## Supplementary Information

**Additional file 1: Figure S1.** Higher neutrophils burden in the epithelial lining fluid (ELF) of alpha1- antitrypsin deficient individuals as compared to control subjects. **(A)** The cell count of neutrophils, **(B)** the percentage of neutrophils in the ELF of alpha1- antitrypsin deficient individuals during one year of follow up (4 visits), and **(C)** the concentrations of NE- alpha1- antitrypsin complexes. * P < 0.05.

## Data Availability

The datasets and analysis of this study are available from the corresponding author on reasonable request.

## Abbreviations

AAT: Alpha-1-antitrypsin
AATD: Alpha-1-antitrypsin deficiency
ANEC: Anti-neutrophil elastase capacity
BALF: Bronchoalveolar lavage fluid
COPD: Chronic obstructive pulmonary disease
CS: Cigarette smoke
∆FEV_1_: Decline in forced expired volume in 1 s
ELF: Epithelial lining fluid
FEV_1_: Forced expired volume in 1 s
IHC: Immunohistochemistry
NE: Neutrophil Elastase
PR3: Proteinase 3
